# Clinical impact of the rapid molecular detection of RSV and influenza A and B viruses in the emergency department

**DOI:** 10.1371/journal.pone.0274222

**Published:** 2022-09-02

**Authors:** Nicolas Yin, Marc Van Nuffelen, Magali Bartiaux, Thierry Préseau, Inge Roggen, Sabrina Delaunoy, Bhavna Mahadeb, Hafid Dahma, Laurent Busson, Olivier Vandenberg, Marie Hallin

**Affiliations:** 1 Department of Microbiology, Laboratoire Hospitalier Universitaire de Bruxelles–Universitair Laboratorium Brussel (LHUB-ULB), Université Libre de Bruxelles (ULB), Brussels, Belgium; 2 Emergency Department, Erasme University Hospital, Université Libre de Bruxelles (ULB), Brussels, Belgium; 3 Emergency Department, Saint-Pierre University Hospital, Université Libre de Bruxelles (ULB), Brussels, Belgium; 4 Emergency Department, Brugmann University Hospital, Université Libre de Bruxelles (ULB), Brussels, Belgium; 5 Emergency Department, Queen Fabiola Pediatric University Hospital, Université Libre de Bruxelles (ULB), Brussels, Belgium; 6 Clinical Research and Innovation Unit, Laboratoire Hospitalier Universitaire de Bruxelles–Universitair Laboratorium Brussel (LHUB-ULB), Université Libre de Bruxelles (ULB), Brussels, Belgium; 7 Centre for Environmental Health and Occupational Health, School of Public Health, Université Libre de Bruxelles (ULB), Brussels, Belgium; 8 Division of Infection and Immunity, Faculty of Medical Sciences, University College London, London, United Kingdom; Defense Threat Reduction Agency, UNITED STATES

## Abstract

**Introduction:**

Using respiratory virus rapid diagnostic tests in the emergency department could allow better and faster clinical management. Point-of-care PCR instruments now provide results in less than 30 minutes. The objective of this study was to assess the impact of the use of a rapid molecular diagnostic test, the cobas® Influenza A/B & RSV Assay, during the clinical management of emergency department patients.

**Methods:**

Patients (adults and children) requiring admission or suffering from an underlying condition at risk of respiratory complications were prospectively recruited in the emergency department of four hospitals in the Brussels region. Physicians’ intentions regarding admission, isolation, antibiotic, and antiviral use were collected before and after performing the rapid molecular test. Additionally, a comparison of the analytical performance of this test against antigen rapid tests and viral culture was performed as well as a time-to-result evaluation.

**Results:**

Among the 293 patients recruited, 90 had a positive PCR, whereas 44 had a positive antigen test. PCR yielded a sensitivity of 100% for all targets. Antigen tests yielded sensitivities ranging from 66.7% for influenza B to 83.3% for respiratory syncytial virus (RSV). The use of PCR allowed a decrease in the overall need for isolation and treatment by limiting the isolation of negative patients and antibiotic use for positive patients. Meanwhile, antiviral treatments better targeted patients with a positive influenza PCR.

**Conclusion:**

The use of a rapid influenza and RSV molecular test improves the clinical management of patients admitted to the emergency department by providing a fast and reliable result. Their additional cost compared to antigen tests should be balanced with the benefit of their analytical performance, leading to efficient reductions in the need for isolation and antibiotic use.

## Introduction

The COVID-19 pandemic has highlighted the need for rapid and trustworthy diagnostic tests for respiratory tract infections to assess patients’ potential infectiousness and to set containment measures [1, 2]. Various studies have previously demonstrated that a fast and reliable diagnosis of respiratory infections improves the clinical management during seasonal epidemics of respiratory syncytial virus (RSV) and influenza A and B viruses [3, 4]. In Europe, before the COVID-19 pandemic, universal health coverage rarely assured access to molecular diagnostic testing for these infections, limiting their prescription in favor of less expensive antigen tests. In Belgium, for instance, SARS-CoV-2 PCR detection is now reimbursed, but for RSV and influenza A and B viruses, only antigen detection is covered [5]. However, compared to RT‒PCR, the sensitivity of these antigen rapid diagnostic tests was estimated to be as low as 53.9% in adults and 64.6% in children regarding influenza A [6] and 74% in children regarding RSV [7]. Furthermore, clinical judgment, followed by PCR or point-of-care testing (POCT), was found to be cost-effective in a setting where influenza probability was high [8]. Fast “sample-in, result-out” PCR instruments, such as the Roche cobas® Liat®, are now providing point-of-care PCR results in less than 30 minutes (a run on the instrument is completed in 21 minutes once the sample is loaded; the hands-on time is approximately 5 minutes) [9]. The objective of this study was to assess the impact of the use of the cobas® Liat® Influenza A/B & RSV assay on the clinical management of emergency department (ED) patients requiring admission or presenting an underlying condition at risk of respiratory complications.

## Material and methods

### Population and data collection

Patients attending the EDs of four hospitals located in the Brussels area of Belgium were prospectively recruited. To be included, patients needed to meet at least one of the following inclusion criteria: (i) respiratory symptoms and a pretest indication of hospitalization by the physician; (ii) respiratory symptoms and an underlying condition at risk of respiratory complication following influenza infection (as described by the European Centre for Disease Prevention and Control and the US Centers for Disease Control: age ≥ 65 years old or < 2 years old, pregnancy, chronic medical conditions, immunocompromised patient, etc. [10, 11]); (iii) age < 3 months with unexplained fever. This study did not use any exclusion criteria. When including the patient and prescribing the test, physicians indicated their intentions regarding antimicrobial treatment, isolation, and hospitalization by answering a questionnaire. The same questionnaire was completed again once the PCR results were available to evaluate the posttest intentions. Written informed consent was collected from each participant or their guardian. The ethics committee of each hospital approved the study.

### Specimen collection and analyses

The accepted samples were nasopharyngeal swabs in 3 mL of universal transport medium (UTM). Nasopharyngeal aspirates diluted with 3 mL of UTM were also accepted for children. Approximately 200 μL was immediately used to perform a rapid PCR using the cobas® Liat® Influenza A/B & RSV assay (Roche Diagnostics, Indianapolis, IN) following the manufacturer’s instructions in a 24/7 on-site laboratory or in a point-of-care setting. PCR results were instantly transmitted to the result servers. For PCRs performed at on-site laboratories, PCR time-to-result was defined as the time between the prescription and the availability of the results. In addition, antigen rapid diagnostic tests (RDTs) were performed after PCR using the Influ A+B K-Set (Coris BioConcept, Gembloux, Belgium) for influenza A and influenza B detection and RSV K-SeT (Coris BioConcept) for RSV detection on the same sample (200 μL/test). Upon arrival in the virology laboratory, 1 mL of UTM was also inoculated onto confluent Vero (African green monkey kidney), MRC5 (human lung) and LLC-MK2 (rhesus monkey kidney) cell cultures (Vircell, Granada, Spain) in 24-well or 6-well tissue culture plates (Greiner-Bio One, Vilvoorde, Belgium). Cultures were incubated at 36°C in a 5% CO_2_ atmosphere for 2 weeks for Vero and LLC-MK2 cells and 3 weeks for MRC5 cells. The media were replaced weekly. Cultures were examined every two to three days using an inverted microscope. Hemadsorption was performed on LLC-MK2 cells at the end of the second week of incubation.

### Statistical analysis

To assess the analytical performance of a molecular detection technique against culture and antigen detection, a composite reference standard was constructed as recommended [12]. Samples considered positive for a viral pathogen were defined as those testing positive for this viral pathogen by at least 2 of the 3 techniques used and negative as those that tested negative by at least 2 of the 3 techniques. Statistical analyses were performed using Analyse-it® for Microsoft Excel v5.30.4 (Analyse-it Software, Leeds, United Kingdom). Proportion variations between pre-test and post-test clinical intentions were evaluated using the McNemar-Mosteller exact test.

## Results

Two hundred and ninety-three patients were recruited, including 68 children (< 15 years old) (Table 1), from February to March 2020. Among them, 71 (incl. 25 children) had a positive rapid PCR result for influenza A only, 10 (incl. 2 children) for influenza B only, 1 child for both influenza A and B, 8 (incl. 5 children) for RSV and 203 (incl. 35 children) were negative for all targets. The positive agreement between PCR and RDT ranged from 36.3% (4/11) for influenza B to 62.5% (5/8) for RSV. One sample was positive for influenza A using RDT although negative by culture and PCR. Likewise, one sample was positive for influenza A using culture although negative by PCR and RDT. Thus, using the composite reference standard, PCR reached a sensitivity of 100% for the 3 viruses targeted (Table 2).

**Table 1:** Population characteristics.

Age (years)	Female	Male	Overall
Overall	123	170	293
< 15	25	43	68
15–65	63	63	126
> 65	35	64	99

**Table 2:** Analytical performance (Se: Sensitivity, Sp: Specificity) and Wilson 95% confidence interval (CI) of the Cobas® Liat Influenza A/B & RSV assay (PCR), antigen tests (RDT) and viral culture for the diagnosis of influenza A, B and RSV using a composite reference standard (samples considered positive or negative if tested as such by at least 2 of the 3 techniques used).

		Reference			
Test	Result	Positive	Negative	Total	Performance (95% CI)
Influenza A					
• PCR	Positive	49	23	72	Se = 100% (92.7–100%)
	Negative	0	221	221	Sp = 90.6% (86.3–93.6%)
• RDT	Positive	34	1	35	Se = 69.4% (55.5–80.5%)
	Negative	15	243	258	Sp = 99.6% (97.7–99.9%)
• Culture*	Positive	42	1	43	Se = 87.5% (75.3–94.1%)
	Negative	6	241	247	Sp = 99.6% (97.7–99.9%)
Influenza B					
• PCR	Positive	6	5	11	Se = 100% (61.0–100%)
	Negative	0	282	282	Sp = 98.3% (96.0–99.3%)
• RDT	Positive	4	0	4	Se = 66.7% (30.0–90.3%)
	Negative	2	287	289	Sp = 100% (98.7–100%)
• Culture*	Positive	6	0	6	Se = 100% (61.0–100%)
	Negative	0	284	284	Sp = 100% (98.7–100%)
RSV					
• PCR	Positive	6	2	8	Se = 100% (61.0–100%)
	Negative	0	285	285	Sp = 99.3% (97.5–99.8%)
• RDT	Positive	5	0	5	Se = 83.3% (43.6–97%)
	Negative	1	287	288	Sp = 100% (98.7–100%)
• Culture*	Positive	5	0	5	Se = 100% (56.6–100%)
	Negative	0	285	285	Sp = 100% (98.7–100%)

*3 RDT-positive/PCR-positive samples were accidentally not transferred to the central laboratory for culture and are therefore missing without impacting the reference standard as they were already concordant.

Clinical intentions regarding admission were not influenced by the result of the test (Table 3). However, an overall decrease in the intentions of admission in isolation (odds ratio (OR) = 0.381; 95% confidence interval (CI) = 0.200–0.692)), antibiotic use (OR = 0.486; CI = 0.261–0.876) and oseltamivir use (OR = 0.543; CI = 0.320–0.903) was observed. These trends were the result of both a sharp decrease in isolation intentions for people with a negative PCR (OR = 0.077; CI = 0.015–0.242) and an increase in isolation intentions for people with a positive PCR (OR = 4.333; CI = 1.191–23.707). Similarly, patients with a positive PCR were less likely to be prescribed antibiotics (OR = 0.235; CI = 0.058–0.721), and oseltamivir treatment was more specifically prescribed for patients with an influenza-positive PCR (OR = 12.500; CI = 3.117–108.889), including for patients with a positive PCR but negative RDT results (OR = 8.500; CI = 2.018–75.851). Positive RDT alone would not have resulted in a significant increase in isolation (OR = +∞; CI = 0.660 –+∞).

**Table 3:** Clinical impact of the positive (+) and negative (-) results of the PCR and antigen rapid diagnostic test (RDT) for respiratory syncytial virus (RSV) and influenza A and B viruses (flu).

Intention	Pre-test	Post-test	Odds ratio	p value*
			(95% confidence interval)	
Admission	175/261	172/261	0.850 (0.418–1.708)	0.74
• PCR +	45/78	41/78	0.429 (0.072–1.877)	0.34
• RDT +	19/37	18/37	0.667 (0.056–5.820)	1.00
• PCR -	130/183	131/183	1.077 (0.470–2.488)	1.00
Isolation**	78/148	52/148	0.381 (0.200–0.692)	0.0009
• PCR +	25/38	35/38	4.333 (1.191–23.707)	0.0213
• PCR +, RDT -	13/22	19/22	3.000 (0.749–17.228)	0.1460
• RDT +	12/16	16/16	+∞ (0.660 –+∞)	0.1250
• PCR -	53/110	17/110	0.077 (0.015–0.242)	<0.0001
• RDT -	66/132	36/132	0.286 (0.137–0.553)	<0.0001
Antibiotic use	117/249	98/249	0.486 (0.261–0.876)	0.0145
• PCR +	30/75	17/75	0.235 (0.058–0.721)	0.0072
• PCR +, RDT -	15/38	9/38	0.333 (0.058–1.336)	0.1460
• RDT +	15/37	8/37	0.125 (0.003–0.932)	0.0391
• PCR -	87/174	81/174	0.700 (0.327–1.457)	0.3915
• RDT-	102/212	90/212	0.586 (0.302–1.103)	0.1038
Oseltamivir use	61/245	40/245	0.543 (0.320–0.903)	0.0170
• PCR flu +	14/67	37/67	12.500 (3.117–108.889)	<0.0001
• PCR flu +, RDT -	7/36	22/36	8.500 (2.018–75.851)	0.0007
• RDT flu +	7/31	15/31	+∞ (1.707 –+∞)	0.0078
• PCR flu -	47/178	3/178	0.000 (0.087–0.087)	<0.0001

*McNemar-Mosteller exact test

**among those with both a pre- and post-test admission intention

Two hundred and eighty-four (97.3%) samples were analyzed in a 24/7 on-site laboratory, and the remaining samples were analyzed in the ED as a point of care. The median time-to-result of the PCR in the laboratory was 60 minutes (CI = 53–76 minutes), with a 90^th^ percentile of 164 minutes.

## Discussion

Previous studies have underscored the impact of the rapid detection of influenza and RSV in the management of patients [13–16]. We showed that an overall significant decrease in antibiotic prescription could be obtained by providing a relevant diagnostic tool combining speed and sensitivity. The excellent sensitivity of the cobas® Liat® assay allowed an optimized assessment of the need for isolation for the inpatients, ensuring proper isolation for almost all infected patients, which likely led to a reduction in nosocomial transmission. Indeed, less than half of the patients with a positive PCR also had a positive RDT, emphasizing the importance of using a molecular test efficiently to improve antibiotic and antiviral stewardship while offering a real assessment for the need for costly hospitalization in isolation. The median days since symptom onset was 2 for RDT-positive patients, whereas it was 2.5 for PCR-positive patients, although the difference was not statistically significant. RDTs are well known to be more sensitive for higher viral loads and hence more infectious patients. Nevertheless, recruited patients were symptomatic; thus, better performance is expected from clinicians for a positive diagnosis regardless of the number of days since symptom onset. The overall decrease in the isolation indications for patients with a negative test should be balanced with the clinical presentation of the patient to prevent the nosocomial transmission of other respiratory pathogens. Nevertheless, in a pandemic context where isolation resources can be scarce, the use of a negative reliable test to decrease the need for isolation should be considered.

In our study, the median time of response from the laboratory was 60 minutes for a less than 30-minute assay. Nevertheless, the time-to-result in this setting is dependent on the overall workload in the on-site laboratory. Using this assay as a POCT at the ED would certainly decrease the time-to-result while increasing the autonomy of the ED team regarding the general management of the workflow and the real-time adaptation of the indications, depending on the current level of bed occupancy and the epidemic situation.

However, such a strategy has a cost and should be further examined through a medico-economic study to better determine the optimal target population while taking into account the subsequent decrease in nosocomial outbreaks and the targeted use of individual protection equipment and antibiotics. In a Dutch study, the daily direct cost of isolating patients ranged from €28 (£23/$31) to €41 (£34/$46) [17]. At the time of writing, the price of the cobas® Liat® Influenza A/B & RSV was 39€/test (£33/$44) (excluding instruments and workforce). In Belgium, an antigen test is reimbursed 8.06€ (£6.74/$9.00) per target (maximum 3) and a viral culture, 45.17€ (£37.75/$50.48). Decreasing the number of incorrectly isolated patients would likely balance the costs of performing PCR instead of RDTs and viral cultures for inpatients during seasonal epidemics.

Even as we were able to evaluate the cobas® Liat® Influenza A/B & RSV assay in the laboratory, this study was prematurely interrupted by the surge of the COVID-19 pandemic in Belgium in March 2020. This event prevented us from evaluating the benefit of the use of this assay as a POCT because this assay was not relevant for the epidemic at the time. The need for human and technical resources also prevented us from cross checking PCR-only positive tests with another molecular method, likely underestimating the specificity of this assay. Likewise, we used a composite reference standard to balance the well-known lower sensitivity of RDT and culture. In the beginning of 2020, the COVID-19 pandemic was associated with smaller outbreaks of influenza and RSV. The positivity rate was lower than that in a previous study [9] and could be an additional limitation. Following the emergence of COVID-19, the implementation of the cobas® Liat® as a POCT in the ED raised biosafety concerns. This assay requires pipetting UTM to the reagent tube, which can be considered a risk of projection and aerosolization. To solve this, the operator performing the sampling wearing personal protection equipment loaded the reagent tube with UTM at the bed side. The sealed tube was then transported to the instrument located in a separate room [18].

In conclusion, using a rapid molecular assay, such as the cobas® Liat® Influenza A/B & RSV, improves the clinical management of patients by refining the indications of isolation, antibiotic, and antiviral treatment due to better analytical performances than RDTs. Using this instrument as a POCT in the ED could provide an efficient 24/7 solution to dispatch inpatients and quickly and optimally manage at-risk outpatients.

## Supporting information

S1 Data (XLSX)
